# Transcellular Transport of Heparin-coated Magnetic Iron Oxide Nanoparticles (Hep-MION) Under the Influence of an Applied Magnetic Field

**DOI:** 10.3390/pharmaceutics2020119

**Published:** 2010-04-26

**Authors:** Kyoung Ah Min, Faquan Yu, Victor C. Yang, Xinyuan Zhang, Gus R. Rosania

**Affiliations:** 1 Department of Pharmaceutical Sciences, College of Pharmacy, University of Michigan, 428 Church St., Ann Arbor, MI 48109, USA; E-Mails: minkah@umich.edu (K.A.M.); fyuwucn@gmail.com (F.Y.); vcyang@umich.edu (V.C.Y.); xinyuan@umich.edu (X.Z.); 2 Key Laboratory for Green Chemical Process of Ministry of Education, Wuhan Institute of Technology, Wuhan 430073, China; 3 Tianjin Key Laboratory for Modern Drug Delivery and High Efficiency, Tianjin University, Tianjin 300072, China

**Keywords:** magnetic iron oxide nanoparticles (MION), magnetic field, transcellular transport, MDCK cell monolayer, drug targeting

## Abstract

In this study, magnetic iron oxide nanoparticles coated with heparin (Hep-MION) were synthesized and the transcellular transport of the nanoparticles across epithelial cell monolayers on porous polyester membranes was investigated. An externally applied magnetic field facilitated the transport of the Hep-MION across cell monolayers. However, high Hep-MION concentrations led to an increased aggregation of nanoparticles on the cell monolayer after application of the magnetic field. Our results indicate that magnetic guidance of Hep-MION most effectively promotes transcellular transport under conditions that minimize formation of magnetically-induced nanoparticle aggregates. Across cell monolayers, the magnet’s attraction led to the greatest increase in mass transport rate in dilute dispersions and in high serum concentrations, suggesting that magnetic guidance may be useful for *in vivo* targeting of Hep-MION.

## 1. Introduction

Magnetic iron oxide nanoparticles (MION) in colloidal dispersions have many different applications, including *in vitro* cell separation [1,2,3], drug delivery [4], gene delivery (*i.e.*, magnetofection) [5,6], tumor hyperthermia [7], and as magnetic resonance imaging (MRI) agents to enhance contrast of organs and tissues [8,9,10]. Magnetic nanoparticles have raised a considerable amount of interest amongst pharmaceutical scientists, as vehicles to deliver genes or drug molecules to specific target sites. The transport of gene or drug molecules to specific target sites can be enhanced by the interaction of magnetic iron oxide nanoparticles with applied magnetic fields [11,12]. When therapeutics (drugs or genes) attached to the MION are injected at or near a target site and an external magnetic field applied, the therapeutic agents can be effectively concentrated in the target cells or tissues [13,14,15]. Therefore, targeted drug or gene delivery with high efficacy and low side effects can be enhanced by using MION. 

Mechanistically, various transport routes can be exploited for directing nanoparticles to specific sites of action within the living organism [16,17]. Epithelial cell monolayers are amongst the most important barriers limiting nanoparticle diffusion and distribution in the body. While lipophilic small molecules can easily diffuse across the plasma membranes of epithelial cells, molecules with larger size and less lipophilicity are not able to cross phospholipid bilayers [18,19]. Instead, they may be able to cross cell monolayers through gaps that may be present between cells, referred to as paracellular transport. We considered the possibility that transport of MION across cell monolayers may be facilitated by an external magnet field by attracting the particles and facilitating their passage between the cells. Transcellular transport of large hydrophilic molecules can be facilitated transporters, adsorptive or receptor-mediated transcytosis, which would be enhanced by high local concentrations of nanoparticles [20,21,22]. Paracellular transport could also be facilitated simply by increasing the concentration of particles at the surface of the cells. 

However, magnetic nanoparticle suspensions also have concentration-dependent stability issues, especially in the presence of a magnetic field. In the presence of a magnetic field, particles tend to attract to each other via strong magnetic dipole-dipole attractions between the particles [12,23]. Anisotropy of the forces between induced dipoles has been reported to cause the particles in dispersions to form linear chains [24]. Moreover, the magnitude of the induced dipolar magnetic forces depends on the intensity of the applied magnetic field. When the magnetic nanoparticles orient in the direction created by the applied magnetic field, the induced dipole-dipole interactions become larger resulting in increased particle aggregation. To optimize these properties, various synthetic methods have been developed to modulate the physicochemical properties of magnetic nanoparticles such as size, charge, and magnetic behavior [25,26,27]. Surface coating methods have been developed to modify the nanoparticles with nontoxic and biocompatible stabilizers for practical biomedical applications of MION. Various polymeric coating materials such as dextran, carboxydextran, starch, and PEG (polyethylene glycol), *etc.* could be useful to prevent irreversible aggregation MION in aqueous or biological media.

In this study, the ferrite cores (maghemite (Fe_2_O_3_), magnetite (Fe_3_O_4_)) of MION coated with polymeric shells containing heparin were synthesized and evaluated in terms of the magnetization by applied magnetic field and transport across semipermeable membranes and epithelial cell monolayers. For testing, the Madin-Darby canine kidney (MDCK) epithelial cell line was used in the transport studies because they can differentiate into polarized columnar epithelium and form the cell monolayer with tight junctions when cultured on permeable membrane supports [28,29,30]. MDCK cells are a common, model cell system to study passive and active, transcellular and paracellular transport mechanisms [29,30,31]. We examined the superparamagnetic properties and stability of the Hep-MION suspensions. Then, we evaluated the transcellular transport of the Hep-MION in the presence and absence of an applied magnetic field. With the *in vitro* cell culture system, we studied how the applied magnetic field modulated the interactions of MIONs and cell monolayers. With microscopic observations, we monitored how the magnetic field affected the aggregation of particles in suspension and at the cell surface. We report that the ability of magnetic field to promote transport was dependent on the concentration of nanoparticles, and was inhibited by the formation of particle aggregates at increasing particle concentrations.

## 2. Experimental Section

### 2.1. Materials

Chemicals used to prepare the iron oxide nanoparticles were ferrous chloride tetrahydrate (Fluka), iron chloride hexahydrate (Sigma-Aldrich), and heparin sodium salt (Sigma, H4784). Lucifer Yellow (LY) was obtained from Sigma-Aldrich and DYNAL®-MPC-L magnet bar was purchased from Invitrogen (Carlsbad, CA). Transwell inserts with polyester membrane (pore size: 3 μm) were obtained from Corning Life Sciences (Lowell, MA). Dulbecco’s Modified Eagle Medium (DMEM), Penicillin-Streptomycin, Dulbecco’s phosphate buffered saline (DPBS), Fetal bovine serum (FBS), and Trypsin-EDTA solution were purchased from Gibco BRL (Invitrogen, Carlsbad, CA). All the chemicals used for preparation of Hank’s balanced salt solution (HBSS) were purchased from Sigma-Aldrich (St. Louis, MO) and Fisher Scientific Co. (Pittsburgh, PA). 

### 2.2. Synthesis of the Hep-MION

MION were synthesized according to the procedure previously reported by Kim *et al.* [32]. The solution containing 0.76 mol/L ferric chloride and 0.4 mol/L of ferrous chloride (molar ratio of ferric to ferrous = 2:1) prepared at pH 1.7 under N_2_ protection was added into a 1.5 M NaOH solution under mechanical stirring. The mixture was gradually heated (1 ºC/min) to 78 ºC and held at this temperature for 1 h with stirring and N_2_ protection. After the supernatant was removed by a permanent magnet, the wet sol was treated with 0.01 M HCl and sonicated for 1 h. The colloidal suspension of MION was filtered through a 0.45 µm and then a 0.22 µm membrane, followed by adjusting to a suspension containing 0.7 mg Fe/mL. Then, 200 mL of 0.7 mg Fe/mL iron oxide nanoparticles were added to 200 mL of 1 mg/mL glycine under stirring condition, ultrasonicated for 20 min, and in further stirred for 2 hours. After free glycine was removed by ultrafiltration, the iron concentrations of the samples were measured by the inductively coupled plasma-optical emission spectroscopy (ICP-OES) analysis using the Perkin-Elmer Optima 2000 DV device (Perkin-Elmer, Inc., Boston, MA, USA), and then diluted to a concentration of 0.35 mg Fe/mL. As a final process, 100 mL of 0.35 mg Fe/mL of glycine-MION were added to 100 mL of 1 mg/mL heparin solution, under stirring condition and ultrasonication. The heparin-coated MION (Hep-MION) were obtained after free heparin was removed by ultrafiltration. 

### 2.3. Physicochemical characterization of the Hep-MION

Volume-weighted size and zeta potential of Hep-MION were measured with a NICOMP 380 ZLS dynamic light scattering (DLS) instrument (PSS, Santa Barbara, CA, USA), using the 632 nm line of a HeNe laser as the incident light. Transmission electron microscopy (TEM) using a JEOL 3011 high-resolution electron microscope (JEOL Tokyo, Japan) at an accelerated voltage of 300 kV. Samples were prepared by placing diluted particle suspensions on formvar film-coated copper grids (01813-F, Ted Pella, Inc, USA) and then dried at room temperature. Superparamagnetic properties of the Hep-MION were examined by using a superconducting quantum interference device (SQUID) (Quantum Design Inc., San Diego, CA, USA) at 25 ºC. The contents of irons in the magnetic nanoparticles were measured by the ICP-OES analysis and calibrated with an internal standard Yttrium and a work curve of iron standard samples from GFS Chemicals®. In order to test stability of the nanoparticles in the transport buffer, the sizes and distributions of nanoparticles in the various solutions such as water, HBSS with 1% FBS, or HBSS with 10% FBS were measured after incubation at 37 ºC using NICOMP 380 ZLS DLS instrument. These nanoparticles solutions were also examined with a Nikon TE2000 inverted microscope (10 × objectives). 

### 2.4. Transport of the Hep-MION across the polyester membrane

For the transport experiments using Hep-MION, the transport buffer, HBSS was prepared containing 137 mM NaCl, 5.4 mM KCl, 0.34 mM Na_2_HPO_4_·7H_2_O, 0.44 mM KH_2_PO_4_ (anhydrous), 1.3 mM CaCl_2_·2H_2_O, 0.8mM MgSO_4_·7H_2_O, 25 mM D-glucose in 1 L of Milli-Q water. As a buffering agent, 10 mM HEPES (4-(2-hydroxyethyl)-1-piperazine ethane sulfonic acid; C_8_H_18_N_2_O_4_S) was added to HBSS. The pH value of the HBSS with 10 mM HEPES was adjusted to be 7.4 by adding 1 N NaOH, and then used for the transport experiments after filtering through a 0.22 μm membrane. The transport experiments of Hep-MION were performed in 24-well culture plates with Transwell inserts of pore size 0.4 or 3 μm with sterile water or HBSS (pH 7.4) in the presence or absence of 1% or 10% FBS. Transport buffer (600 µL) at 37 ºC without the nanoparticles was added into the receiver chambers (basolateral compartments) in 24-well plates. The nanoparticle solution (100 µL of Hep-MION in transport buffer; 0.206, 0.2575, or 0.412 mg/mL) was added to the donor chamber (apical compartment) in Transwell insert with the polyester membrane. The insert containing the nanoparticle solution was transferred to the next well containing 600 µL of fresh transport buffer without the nanoparticles at each time point. After transport experiments were performed under stirring condition with VWR rocking platform shakers, the sample solutions were collected from the receiver (basolateral) sides at each time point and the donor (apical) side at the last time point. The solutions that might contain the remaining nanoparticles were also taken by washing the walls of the donor sides and receiver sides with the transport buffer. Standard solutions for various concentration ranges (0.000515, 0.00103, 0.00206, 0.00412, 0.0103, 0.0206 mg/mL) were made by diluting the nanoparticle stock solution in sterile water (2.06 mg/mL) with the transport buffer. Two hundred μl of the standard solutions and all the samples were put into each well of Costar® 96-well cell culture plates (Corning Life Sciences), and the UV absorbance of the solutions in each well was measured at 364 nm by the UV plate reader (Powerwave 340, BioTEK, co.). The concentrations of the nanoparticles in all the samples were calculated based on this standard curve. The transcellular permeability coefficient, *P_eff_* (cm/sec) was calculated by normalizing the mass transport rate (*dM/dt*) with the insert area, *A_insert_* (0.33 cm^2^) and apical concentration of nanoparticles, *C_ap_* as shown in the following equation (1):

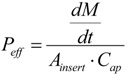
(1)

With the magnet bar located beneath the plates, the transport experiments with shaking were also performed in order to assess the mass transport of nanoparticles in the presence of an applied magnetic field. 

### 2.5. Transport of the Hep-MION across the cell monolayer

MDCK strain II cells obtained from American Type Culture Collection (ATCC) (Manassas, VA) were maintained in 75-cm^2^ flasks at 37 ºC in a 5% CO_2_ containing humidified incubator. MDCK cells were cultured with media containing the DMEM with 2 mM L-glutamine, 4500 mg/L of D-glucose, and 110 mg/L of sodium pyruvate, 1× non-essential amino acids (Gibco 11140), 1% penicillin-Streptomycin (Gibco 10378), and 10% FBS. Culture media were changed every second day during the cultures. At confluency, MDCK cells were trypsinized from the culture flasks and resuspended in the media. One hundred μL of the cell suspension with the density of 4 × 10^5^ cells/cm^2^ was added on the apical side of polyester membrane (0.33 cm^2^, Transwell inserts, pore size: 3 μm) in 24-well culture plate containing 600 μL of media. After overnight incubation at 37 ºC in a 5% CO_2_ atmosphere, the cell morphology and confluency were determined with the Nikon TE2000 microscope. Confluent cell monolayers seeded on transwell inserts in 24-well plates were rinsed twice by HBSS without the nanoparticles and incubated for 20 min in the HBSS with 10% FBS at 37 ºC in a 5% CO_2_ atmosphere. After the incubation, transepithelial electrical resistance (TEER) across the cell monolayer was measured at room temperature in each insert with the cells by Millipore Millicell® ERS electrodes. The inserts with the cells with TEER values higher than 150 Ω× cm^2^ after 1 day’s incubation was used for the transport experiments. All the transport experiments with or without the magnetic field were performed in the same way as described in the Experimental Section 2.4. The apical-to-basolateral transport experiments were conducted until 90 min with the magnetic bar (DYNAL®-MPC-L), while the experiments without the magnetic bar were performed until 120 min. Transcellular permeability coefficients, *P_eff_* was calculated with the mass transport across the cell monolayer with equations (1). After the experiments, TEER was measured and the cell monolayer was examined with the microscope to verify the integrity of cell monolayer. Permeability of Lucifer Yellow (LY) was also measured with confluent cells on inserrts to examine the intactness of the cell monolayer, using the Perkin-Elmer LS 55 fluorescence spectrometer (Ex 430 nm/Em 520 nm) to measure LY concentration changes in the basolateral compartment (with the aid of a standard curve). As a fluid phase marker, LY was used in order to examine endocytosis, in the presence or absence of nanoparticles, with MDCK cells exposed to magnetic fields [33]. Transport experiments were carried out by adding 80 μL of nanoparticle solution (0.322 mg/mL) and 20 μL of 10 mM Lucifer Yellow in the apical side with or without the magnetic field. After 90 min, the walls of apical side and basolateral side were rinsed twice with DPBS and the cells on the membrane in the insert were detached by trypsinization. The cells were examined in 96-well optical bottom plates on the Nikon TE2000S epifluorescence microscope using a standard FITC filter set acquisition channel (100 × objectives). Images were acquired with a CCD camera (Princeton Instruments). All the cell images were analyzed with Adobe photoshop and Metamorph® software. 

## 3. Results and Discussion

### 3.1. Physicochemical characterization of the Hep-MION nanoparticles

**Figure 1:**
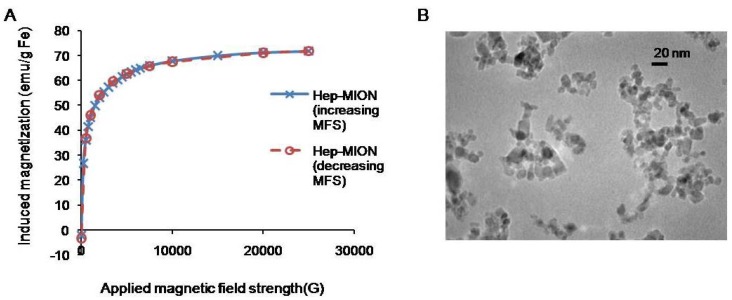
Physicochemical characterization of Hep-MION. **(a)** Magnetization of the Hep-MION was displayed as a function of applied magnetic field (Gauss) in a range between 0 and 30,000 G by using the SQUID at 25 ºC. The magnetization data from SQUID analysis were normalized by Fe content with the unit of emu per gram of Fe. The magnetization curve of the Hep-MION under increasing magnetic field (MFS) overlapped with the demagnetization curve under decreasing magnetic field, consistent with the expected superparamagnetic properties of Hep-MION. **(b)** Transmission electron microscopic images were captured at an accelerated voltage of 300 kV. Scale bar on the image is 20 nm.

Tissue targeting by magnetic nanoparticles depends on the magnetic susceptibility, size distribution and superparamagnetic properties of MION [23,25,26,27]. The magnetization/demagnetization curves of Hep-MION exhibited superparamagnetic behaviors without any hysteresis loop or remanence (Figure 1a). 

With increasing magnetic field (Gauss), the magnetization curves reached a plateau at high magnetic fields. As the applied magnetic field decreased, the Hep-MION became demagnetized and finally had negligible remnant field in the absence of an applied magnetic field. The remnant field of these nanoparticles was almost zero in the absence of an applied magnetic field. Our results were consistent with SQUID magnetization/demagnetization curves of different MION superparamagnetic nanoparticles [34]. The lowered saturation magnetization (70 emu/g Fe) compared to bulk magnetite (92 emu/g Fe) is a phenomenon that is commonly observed with magnetic nanoparticles [35]. This has been typically attributed to the surface effects arising from a magnetically inactive layer covering the ferrite cores (maghemite (Fe_2_O_3_), magnetite (Fe_3_O_4_)). The saturation magnetization has also been shown to be dependent on synthesis methods and also particle size; decreasing as particle size is reduced [36,37]. The hydrodynamic size and zeta potential of the Hep-MION nanoparticles were 40.6 nm (±30 nm) and -51.2 mV, respectively. TEM images showed that these nanoparticles had small sizes around 10 nm which were smaller than the hydrodynamic size measured by light scattering in water (Figure 1b). According to previous studies, MION particles 6–15 nm in diameter can have a single magnetic domain with superparamagnetic properties, so they can be used for effective magnetic targeting or imaging [38]. 

### 3.2. Stability of the Hep-MION nanoparticle dispersions

**Figure 2:**
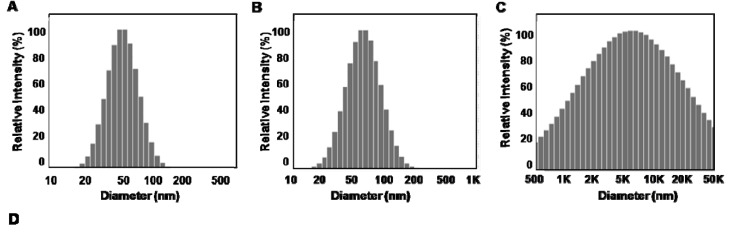
Stability of Hep-MION dispersions in physiological buffers. Dynamic light scattering histograms of Hep-MION in water**(A)**, HBSS with 10% FBS **(B)**, and HBSS with 1% FBS **(C)** after 24 h incubation at 37 ºC. Relative intensity values (%) are displayed with diameters (nm) in a log-scale. Particle size measurement of Hep MIONs **(D)** in three different batches in HBSS with 1% FBS, 10% FBS, or water after incubation at 37 ºC for 0, 5 and 24 h (n = 3). Sizes and distributions of nanoparticles in the various solutions (water, HBSS with 1% FBS, or HBSS with 10% FBS) were measured using NICOMP 380 ZLS DLS instrument after incubation at 37 ºC.

The colloidal dispersions of Hep-MION were stable in water and maintained the hydrodynamic size distribution of the particles at room temperature even after the nanoparticles were stored at 4 ºC more than 1 year. To examine the stability of Hep-MION in physiological conditions, the nanoparticles were diluted in various buffers. Brown precipitates formed in PBS (phosphate buffered saline) without calcium and magnesium ions within 40 min at room temperature (data not shown). Larger precipitates formed in HBSS (cell culture medium containing calcium and magnesium ions). In the presence of 1% fetal bovine serum (FBS) added into HBSS, brown precipitates were visible after 5 h incubation at 37 ºC (data not shown). However, when FBS was added at higher concentrations (10%), the suspension of nanoparticles did not exhibit precipitates, even after 24 h incubation at 37 ºC. Therefore, serum components appeared to stabilize these nanoparticle suspensions. To confirm these observations, hydrodynamic sizes of nanoparticles of different three batches in various solutions were measured after incubation at 37 ºC during different time period (Figure 2). Dynamic light scattering histograms (DLS plots) demonstrate the larger average values of particle size in HBSS containing 1% FBS, compared to the samples in water or HBSS with 10 % FBS (Figure 2a, b, and c) after 24 h incubation at 37 ºC. After 5 h incubation, the average hydrodynamic sizes of the nanoparticles of three different batches in water, HBSS with 10% FBS, or 1% FBS were 57.93 nm (±9.10 nm), 76.70 nm (±7.33 nm), or 3515.53 nm (±260.23 nm), respectively (Figure 2d). The narrow size distribution of nanoparticle suspension in water or HBSS with 10% FBS was not changed during the incubation period, but the particle sizes in HBSS with 1% FBS showed an increase in size and size distribution during the incubation period up to 24 h (Figure 2d) consisting with the formation of aggregates of increasing size. 

By visual inspection, we also determined how magnetic attraction patterns of Hep-MION varied in the presence of an applied external magnetic field (DYNAL®-MPC-L) in various buffer conditions. As time passed under the applied magnetic field, more iron oxide nanoparticles attracted to the magnet were visible as brown precipitates in HBSS with 10% FBS or water. In addition, the reversibility of magnetically-induced nanoparticle aggregates was examined in the capillary tubes in the presence of the applied magnetic field under various buffer conditions. In water or HBSS with 10% FBS, the particles attracted by the magnetic became dispersed again, after the applied magnetic field was removed (data not shown). 

**Table:** 

Buffer	Water			HBSS + 10% FBS			HBSS + 1% FBS		
**Incubation Time (h)**	**0 h**	**5 h**	**24 h**	**0 h**	**5 h**	**24 h**	**0 h**	**5 h**	**24 h**
**Average Size (nm)**	61.37	57.93	59.50	75.47	76.70	76.37	1053.27	3515.53	17659.43
**S.D. (nm)**	6.07	9.10	6.38	6.31	7.33	7.46	66.82	260.23	1243.76

### 3.3. Transport of the Hep-MION across porous membranes

Aggregation of Hep-MION dispersions in HBSS with 1% FBS occurred gradually over a 24 h period (Figure 2c and Table ). Upon 24 h incubation, the measured aggregate size appeared greater than the membrane pore size. To minimize buffer-induced aggregation and establish the effect of magnetic fields on Hep-MION permeability across porous membranes, transport experiments were performed with nucleopore polyester membranes with the pore size of 3 µm within 1 min upon dispersion in transport buffers. In the case of 0.206 mg/mL of nanoparticles in HBSS with 10% or 1% FBS, the *P_eff_* approximately doubled in the presence of the magnetic field. At higher nanoparticle concentrations in the absence of a magnetic field, permeability of Hep-MION was greater in 10% FBS *vs*. 1% FBS (Table 1), suggesting that components in serum are affecting the diffusion behavior of nanoparticles across the membrane pores. With 0.412 mg/mL of nanoparticles in HBSS with 1% FBS, *P_eff_* was 2.4-fold higher in the presence of a magnetic field. In HBSS with 10% FBS, the difference in *P_eff_* in the presence or absence of the magnetic field became smaller with increasing nanoparticle concentrations. Therefore, the effect of the magnetic field on the transport of Hep-MION appears to be a complex function of both the concentration of the particles in the solution, as well as the effect of the medium on the stability of the dispersions and the diffusion of the particles across the membrane pores.

**Table 1:** Comparing the transport behavior of Hep-MION in HBSS with 10% or 1% FBS, with or without the applied magnetic field. Permeability coefficients (*P_eff_*) were assessed with three different concentrations of nanoparticles (0.206, 0.2575 or 0.412 mg/mL) in triplicates. Average values of *P_eff_* are displayed with standard deviations in the parenthesis.

Concentration of Hep-MION	HBSS with 10% FBS		HBSS with 1% FBS	
	Magnet (-)	Magnet (+)	Magnet (-)	Magnet (+)
	*P_eff_* (10^-3^ cm/sec)		*P_eff_* (10^-3^ cm/sec)	
**0.206 mg/mL**	3.19 (0.425)	7.21 (0.53)	3.04 (0.449)	6.77 (1.53)
**0.2575 mg/mL**	5.73 (0.272)	8.47 (0.408)	2.67 (0.544)	6.67 (0.679)
**0.412 mg/mL**	6.72 (0.17)	7.85 (0.849)	2.45 (0.425)	5.88 (0.736)

### 3.4. Transport of the Hep-MION across cell monolayers promoted by a magnetic field

**Figure 3:**
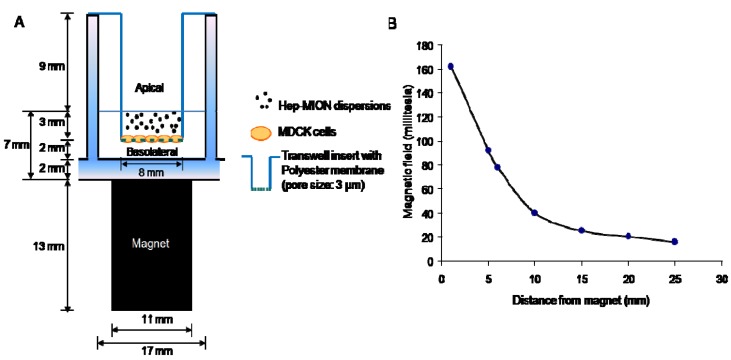
Diagram of the experimental set up. **(A)** The cells were seeded on the polyester membrane (pore size: 3 µm) in Transwell inserts. One magnet (11 mm × 13mm) was placed beneath the well. Hep-MION dispersions were added into the apical compartment (donor chamber) to be transported through the porous membrane into the basolateral compartment (receiver chamber) containing transport buffer. Lengths and widths of the insert in the 24-well plate and magnet are displayed as millimeters. **(B)** Applied magnetic field of the magnet decreases with the increasing distance from the magnet. A 3-axis Hall Teslameter (THM 7025, GMW Associates, San Carlos, CA) was used to measure the magnetic field. X-axis of the graph represents the vertical distance from the magnet’s surface.

**Figure 4:**
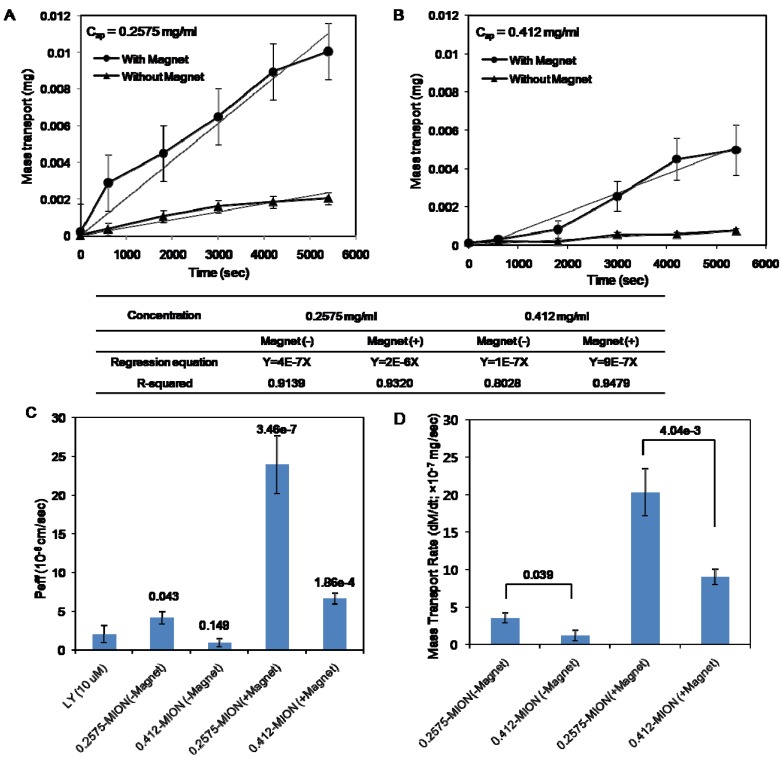
Quantitative analysis of apical-to-basolateral (AP-to-BL) mass transport of Hep-MION across MDCK cell monolayers. Transport experiments across confluent monolayers were performed with Hep-MION dispersions at 0.2575 **(A)** or 0.412 mg/mL **(B)** in HBSS with 10% FBS. Experiments were performed in triplicates and standard error bars are shown. Equations and R^2^ values of the regression lines of mass transport as a function of time for the apical concentration (C_ap_; 0.2575 and 0.412 mg/mL) are displayed in the table. **(C)** The permeability coefficient, *P_eff_* values of the nanoparticles (C_ap_; 0.2575 and 0.412 mg/mL) were compared with the permeability of Lucifer Yellow (LY). P-values of t-test results are indicated over the each bar. **(D)** The mass transport rates (*dM/dt*) of the nanoparticles (C_ap_; 0.2575 and 0.412 mg/mL) were displayed with p-values of t-test results to show the different effects of concentration of Hep-MION on the transcellular transport in the presence or absence of the applied magnetic field.

Next, transcellular transport experiments with Hep-MION were performed using MDCK cells grown on the polyester membrane (pore size: 3 μm). The experimental set up consisted of the cells sitting on the porous membrane in the Transwell insert of 24-well plate, and buffer solutions in apical (donor chamber) and basolateral sides (receiver chamber) (Figure 3a). Applied magnetic field was provided by a permanent magnet at the bottom of the insert. As indicated, the upper boundary of buffer containing Hep-MION dispersions was separated from the surface of the magnet by 7 mm and the distance between the nucleopore membrane and magnet was 4 mm. As the magnetic field was applied from underneath the plate, Hep-MION dispersions were attracted towards the membranes. Based on magnetic field measurements (Figure 3b), the strength of the magnetic field at the level of the cell monolayer (4 mm-distance from magnet) was 100 millitesla while at the upper boundary of the nanoparticle dispersions (7 mm-distance from magnet) was about 65 millitesla. According to this experimental set up, in apical-to-basolateral transport experiments, the permeability coefficients (*P_eff_*) of membranes covered by cell monolayers were about 3 orders of magnitude less than membranes without cells (Table 1 and Table 2). The rate of mass transport of nanoparticles across cell monolayer was greater in the presence than in the absence of the applied magnetic field (Figure 4a and 4b). The nanoparticles with high concentration (0.412 mg/mL) showed much lower mass transport at each time point than the nanoparticles of 0.2575 mg/mL under the applied magnetic field. The calculated permeability and mass transport rate across MDCK cell monolayers increased approximately ten-fold when the transport experiments were conducted in the presence of the applied magnetic field (Table 2). Based on t-test results, the permeability behaviors of iron oxide nanoparticles in the presence of an applied magnetic field were significantly different from those without the magnetic field. Nevertheless, the permeability enhancing effect of the magnetic field was much greater in low concentrations (0.2575 mg/mL) of nanoparticles than higher concentrations (0.412 mg/mL; Table 2). 

**Table 2:** Transport behavior of Hep-MION dispersions (0.2575 or 0.412 mg/mL) with or without an applied magnetic field. The average and standard deviation (S.D.) of three different batches are displayed. The p-values (two-tails) were assessed with two sample t-test with equal variance (significance level: p < 0.05).

	*P_eff_* (10^-6^ cm/sec)				*dM/dt* (10^-7^ mg/sec)			
Concentration (mg/mL)	0.2575		0.412		0.2575		0.412	
**Magnet**	**-**	**+**	**-**	**+**	**-**	**+**	**-**	**+**
**Average**	4.2	24	0.9	6.6	3.5	20	1.2	9
**S.D.**	0.8	3.7	0.5	0.7	0.7	3.2	0.7	1.0
**p-value**	5.81 × 10^-^^3^		3.94 × 10^-4^		5.81 × 10^-^^3^		3.94 × 10^-4^	

As a reference and internal control, we compared the permeability of Hep-MION at two different concentrations (0.2575 and 0.412 mg/mL) with the permeability of Lucifer Yellow (LY; 10 μM) in the same solution. LY is a soluble cell impermeant marker of paracellular transport. The permeability of 0.2575 mg/mL of nanoparticles without a magnetic field was slightly greater than the LY permeability (Figure 4c), while the permeability of 0.412 mg/mL of nanoparticles without magnetic field was similar to LY permeability (p = 0.149). Most remarkably, the permeability of Hep-MION under the applied magnetic field was much greater and statistically different from that of LY permeability (p-value < 10^-^^7^ (0.2575 mg/mL) and < 10^-4^ (0.412 mg/mL); Figure 4c), indicating that the permeability enhancement is due to a specific effect of the magnetic field on the particles. When the mass transport rates (*dM/dt*) were compared for different concentrations of Hep-MION under the same experimental conditions, *dM/dt* in low concentration (0.2575 mg/mL) of Hep-MION was higher than that of high concentration (0.412 mg/mL) of nanoparticles. Although we cannot explain the reason behind this difference it appeared to be statistically significance (Figure 4d; p < 0.05).

### 3.5. Accumulation of Hep-MION on cell monolayers induced by a magnetic field

**Figure 5:**
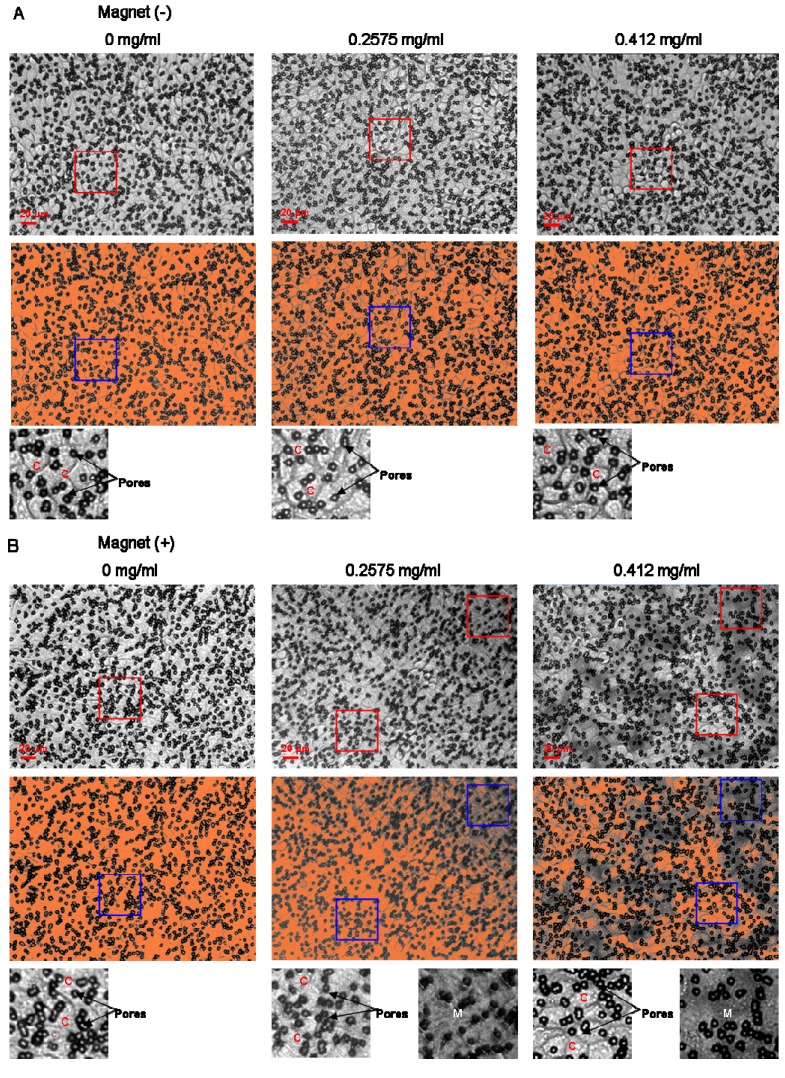
Bright field microscopy of MDCK cell monolayers on polyester membrane (pore size: 3 μm) after transport experiments with Hep-MION. The cell monolayer without **(A)** or with the magnetic field **(B)** is shown (20 × magnifications). Beneath each image of the cell monolayers, the regions of the monolayer that do not exhibit shading due to the dark nanoparticle aggregates are highlighted in orange. Zoom-in regions are indicated with red and blue boxes in the original and highlighted orange images. The dark pores (size: 3 μm) (indicated with arrows labeled “Pores”) are randomly distributed on the membrane and pointed to in the zoom-in images with the cells **(C)**. With an applied magnetic field, the darker regions (in zoom-in images) of the cell monolayer reflect accumulation of magnetic nanoparticles (“M”) in association with the cell monolayer (0.2575 or 0.412 mg/mL). Scale bar corresponds to 20 µm in the bright field images.

After the transport experiments, the cell monolayer on the inserts was examined with a microscope. There were dark regions on the cell monolayer exposed to a magnetic field even after various washing steps, suggesting nanoparticles were strongly bound or internalized by the cells (Figure 5). Without the magnet, the cell monolayer in the treatment group (0.2575 or 0.412 mg/mL) was not different from the control cell monolayer without the nanoparticles (Figure 5a). However, after the transport experiments with the magnet, the cell monolayer in the treatment group became dark with nanoparticles. At high nanoparticle concentrations, nanoparticle aggregates were visible as opaque patches covering the cells (Figure 5b). From these observations (Figure 4 and 5), we cannot distinguish if Hep-MION are transported across the cell monolayer by transcellular or paracellular pathways. In either case, the transcellular transport of Hep-MION at low concentration (0.2575 mg/mL) could be facilitated by the increased concentration of particles at the cell surface. Transcellular transport of Hep-MION at high concentration (0.412 mg/mL) can be enhanced by the magnetic field to some degree, but the effect of the magnet at high concentration seems to be much smaller than at low concentration because particles can form large aggregates, upon interacting with the cells and with each other. 

**Figure 6:**
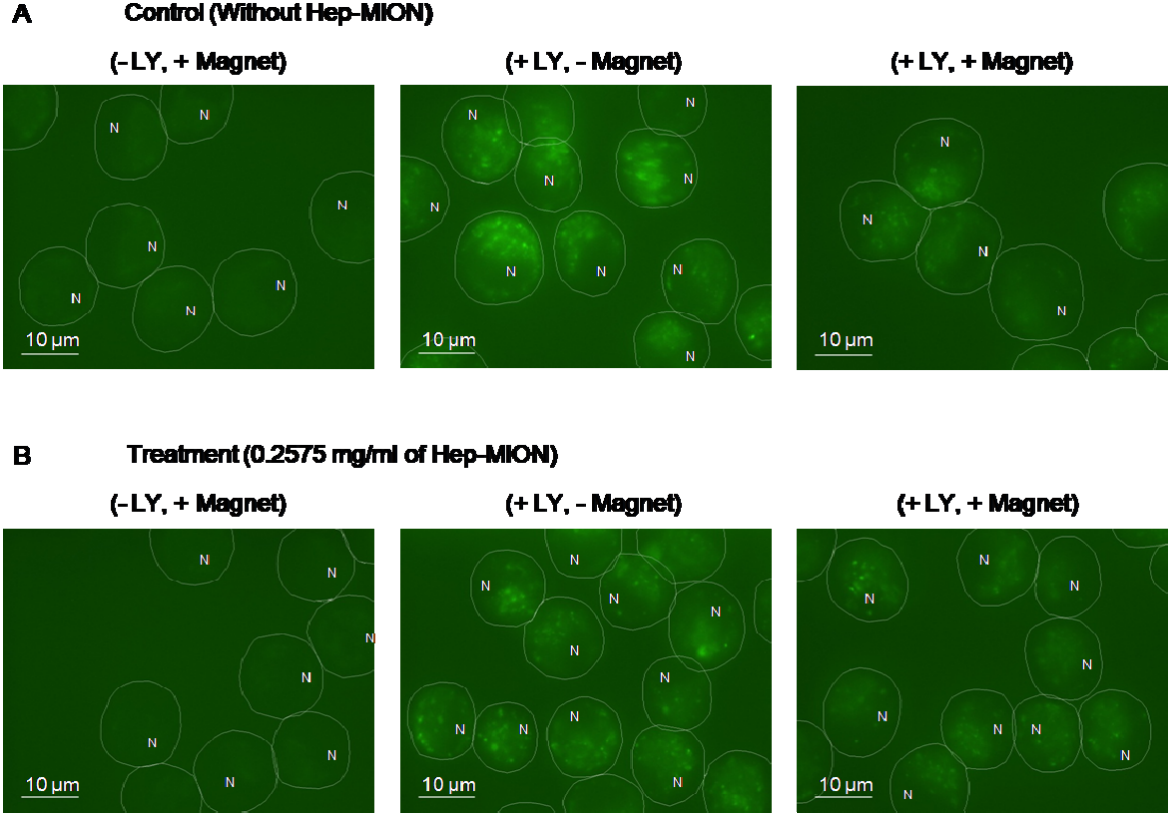
Lucifer Yellow (LY) uptake in the presence and absence of Hep-MION was investigated in transcellular transport experiments, with and without the magnetic field. The vesicles inside the cells became labeled upon incubation with the fluorescent fluid phase marker, LY, visible using the FITC excitation/emission channel of the Nikon TE2000S epifluorescence microscope (100 × objective). **(A)** Control experiments without Hep-MION. **(B)** Experimental treatment group with Hep-MION (0.2575 mg/mL). Scale bar of 10 µm and nucleus of each cell as “N” are displayed in each image. For display, contrast was enhanced and the circumference of each cell (in white) was manually outlined with Adobe Photoshop.

In order to determine whether the magnetically-induced particle associated with cells was specific to magnetized particles, the transport experiments were performed using LY as an internal, soluble reference marker. Cells were exposed to nanoparticles and LY, in the presence and absence of a magnetic field. After the experiments, cells were detached and examined by epifluorescence microscopy (Figure 6). LY served as a fluorescent fluid phase marker and showed endocytosed vesicles inside the cells [33]. The overall size or distributions of LY positive vesicles inside the cells were relatively similar, in the presence and absence of the magnetic field. Therefore the ability of the magnetic field to promote the accumulation of Hep-MION on the cell monolayer is the result of a specific interaction between the magnetic field and the particles, as it does not affect the interaction between the cells and LY, a soluble fluid phase marker of pinocytic uptake.

## 4. Conclusions

Hep-MION is a viable, candidate magnetic carrier for drug targeting or magnetic resonance imaging (MRI). In addition to the observed superparamagnetic properties, these magnetic nanoparticles have narrow size distribution and remain dispersed in physiological medium containing high serum concentrations so their physicochemical and stability properties are consistent with *in vivo* application. Hep-MION at high concentrations can form aggregates, especially in the presence of a magnetic field [39]. In the presence of a porous membrane or a cell monolayer, the diffusion of the particles at the membrane or cell monolayer surface becomes limited, leading to accumulation of particle aggregates at the cell or membrane surface. Accordingly, the effect of a magnetic field on the permeability of particles across porous membranes or cell monolayers is lower at high particle concentrations compared to lower particle concentrations. Low concentration of Hep-MION (0.2575 mg/mL) was less responsive to the magnetic field than higher concentration (0.412 mg/mL), but because the induced aggregates were smaller, the magnetically-induced increase in nanoparticle transport across nucleopore membranes or cell monolayers was relatively greater. 
